# Exposure to secondhand smoke in hospitality settings in Ghana: Evidence of changes since implementation of smoke-free legislation

**DOI:** 10.18332/tid/120934

**Published:** 2020-05-20

**Authors:** Arti Singh, Gabriel Okello, Sean Semple, Fiona Dobbie, Tarja I. Kinnunen, Kwabena F. Lartey, Divine D. Logo, Linda Bauld, Sampson T. Ankrah, Ann McNeill, Ellis Owusu-Dabo

**Affiliations:** 1 School of Public Health, College of Health Sciences, Kwame Nkrumah University of Science and Technology, Kumasi, Ghana; 2 African Centre for Clean Air, Kampala, Uganda; 3 University of Stirling, Stirling, United Kingdom; 4 Usher Institute, University of Edinburgh, Edinburgh, United Kingdom; 5 Faculty of Social Sciences, Tampere University, Tampere, Finland; 6 Department of Mathematics, Kwame Nkrumah University of Science and Technology, Kumasi, Ghana; 7 King’s College London, London, United Kingdom

**Keywords:** observation, hospitality venues, Ghana, air quality monitoring, particulate matter

## Abstract

**INTRODUCTION:**

Ghana has a partial smoking ban with smoking allowed in designated smoking areas. Studies evaluating smoke-free laws are scarce in Sub-Saharan Africa. Evaluation of smoke-free laws is an effective means of measuring progress towards a smoke-free society. This study assessed the level of compliance to the provisions of the current smoke-free policy using air quality measurements for fine particulate matter (PM_2.5_) in hospitality venues in Ghana.

**METHODS:**

This was a cross-sectional observational study conducted in 2019 using a structured observational checklist complemented with air quality measurements using Dylos monitors across 152 randomly selected hospitality venues in three large cities in Ghana.

**RESULTS:**

Smoking was observed in a third of the venues visited. The median indoor PM_2.5_ concentration was 14.6 μg/m^3^ (range: 5.2–349). PM_2.5_ concentrations were higher in venues where smoking was observed (28.3 μg/m^3^) compared to venues where smoking was not observed (12.3 μg/m^3^) (p<0.001). Hospitality locations in Accra, Ghana’s capital city, had the lowest compliance levels (59.5%) and poorer air quality compared to the cities of Kumasi and Tamale.

**CONCLUSIONS:**

The study shows that while smoking and SHS exposure continues in a substantial number of hospitality venues, there is a marked improvement in PM_2.5_ concentrations compared to earlier studies in Ghana. There is still a considerable way to go to increase compliance with the law. Efforts are needed to develop an action plan to build upon recent progress in providing smoke-free public spaces in Ghana.

## INTRODUCTION

Implementing smoke-free legislation remains a challenge in many low- and middle-income countries (LMICs). However, with 77% of all smoking-related deaths and 89% of secondhand smoke (SHS) related-deaths occurring in low- and middle-income countries, it is clear that the burden of the tobacco epidemic has moved from high-income countries (HICs) to LMICs^1^. This means that implementation of smoke-free laws in LMICs is paramount^2^. Article 8 of the World Health

Organization (WHO) Framework Convention on Tobacco Control (FCTC)^3^ and its guidelines including other evidence-based policies such as MPOWER (the WHO’s technical assistance package of evidence-based policies, for more information please see https://www.who.int/tobacco/mpower/mpower_report_six_policies_2008.pdf)^4^ mandate protection from exposure to secondhand smoke (SHS). The WHO African Region also advocates that all countries be compliant with the requirements of FCTC Article 8 guidelines, and that 100% smoke-free environments should become the *status quo* in all societies. This includes hospitality venues (such as bars, hotels, restaurants, night clubs, and pubs) where workers have traditionally been exposed to the highest levels of SHS^5^. While smoke-free policies are becoming more common, more than 80% of the world’s population (particularly in LMICS) is not yet protected by these policies^6,7^. This is the scenario in several countries in Sub-Saharan Africa (SSA) where smoke-free policies either do not exist or are in the inceptive stages and studies on the magnitude of SHS-related air quality are poorly described and inadequate^7^.

Ghana, being one of the first countries to ratify the WHO FCTC in 2004, passed a Tobacco Control Act in 2012 as part of its legal obligation^8^. Section 58 (1) of the Tobacco Control Act prohibits smoking in ‘an enclosed or indoor area of a workplace, or any other public place except in a designated area’. This was later followed by a legislative instrument in 2016 (L.I.2247) that further reiterated smoke-free policies in furtherance to provisions in the tobacco control act and had specific guidelines for setting up designated smoking areas and display of appropriate ‘NO SMOKING’ signage^9^. Thus, Ghana has a partial smoke-free law as smoking is prohibited in enclosed or indoor areas of the workplace or any other public place, with the display of adequate ‘NO SMOKING’ signages posted and ashtrays not displayed, except in a designated smoking area (DSA)^10^. Despite these binding principles, smoking prevalence among the youth (aged 11–17 years) continues to rise (up to 7%) and close to 50% of students are unaware of the harmful effect of SHS^11^. Furthermore, 1 in 10 children are exposed daily to SHS in homes^12^.

Reducing the exposure to SHS is an important public health challenge that has been recognized by policymakers and regulators, and smokers’ behaviour is influenced in part by their understanding of smoke-free legislation. Though the WHO recommends that all countries implement comprehensive smoke-free policies, defined as smoke-free policies with no exemptions for particular venue types or allowances for designated smoking areas, Ghana has a partial smoke-free policy that allows smoking to continue in certain types of enclosed public venues^13^. Effectiveness of comprehensive smoke-free laws have been demonstrated in many countries. For example, in Scotland air quality in bars and pubs was shown to have improved markedly after the introduction of comprehensive smoke-free laws^14^. Similar findings have been demonstrated in England, Wales, Ireland and other HICs^15,16^.

There is limited evidence relating to the evaluation of the current smoke-free law and compliance levels in Ghana. Studies conducted in Ghana pre law (2007) indicated very high levels of SHS exposure (median PM_2.5_ = 553 μg/m^3^) in hospitality venues located in the urban cities of Ghana^17^. A follow up study conducted in 2015 showed similar findings (median PM_2.5_ = 439 μg/m^3^)^18^. Now, more than 5 years into Ghana’s smoke-free policy, it is timely to evaluate the current policy given the rising smoking rates among young people and the use of other tobacco products (such as shisha), in addition to providing comparative data to the previous studies in Ghana^10,19^. Evaluating the law is also useful to identify gaps and check compliance with existing regulations, and in the identification of areas requiring more effective enforcement. This study, therefore, aimed to determine the compliance to the provisions of the current smoke-free policies as identified in the Tobacco Control Act (2012) and L.I (2016) and provide objective data on SHS (by measuring fine particulate matter, PM_2.5_, as a marker of SHS) in hospitality venues.

## METHODS

### Study design

This was a cross-sectional study comprising objective measurements of airborne fine particulate matter (PM_2.5_) in hospitality venues across three cities in Ghana. The measurements were complemented with covert observations of smoking related behavior, signage and compliance with local laws in each venue.

### Training

A team comprising the researcher and four research assistants received training on air-quality monitoring using a low-cost monitor and compliance studies involving observational data collection. Training involved: how to operate a Dylos DC1700 (Dylos Inc, CA, USA) air quality monitor; how to download acquired data; and how to collect data in hospitality venues using an observation checklist protocol similar to that used in studies in similar settings over the past decade^20,21^. The protocol included details on venue selection, visit duration, researcher safety, inside/outside air monitoring duration, logging data, assessment sheet instructions, and data transferring.

### Site selection

The study was conducted in the three largest cities in Ghana; Accra, Kumasi and Tamale (due to their large population density, diversity, and high smoking prevalence). A list of 1532 hospitality venues of bars/pubs/restaurants/hotels and nightclubs in the three cities was obtained from the Ghana tourist authority. These venues were then stratified into the 3 major cities of the southern, middle and northern belts of Ghana, respectively; Kumasi (457), Accra (949), and Tamale (126). Using a margin of error of 5%, confidence limit of 95% and a no-response rate of 87.7%, a total of 154 venues were obtained as the sample size of the study. A proportionate allocation was then done for each of the three cities to gather a convenience sample of 150 venues across the country. A random number generator (Minitab version 17) was then used to randomly select 150 venues from each city. Visits took place during peak working hours (from 16:00 to midnight) in each of the selected cities. In cases where the venue was closed or no longer in operation, the venue next on the list was visited.

### Data collection

A total of 154 venues were visited from the three cities. Data were collected over a 10-week period from July to September 2019 including a three-day pilot data collection in Kumasi. All data collection at the hospitality venues was done in pairs (the researcher and an assistant) on any particular day.

### Covert observations

Observational methods such as visual inspection of a venue (e.g. surveying rooms for the posting of ‘No Smoking’ signs, staff/customer smoking, presence of DSAs, evidence of ashtrays and cigarette butts) and semi-subjective assessment of the presence of recent smoking through self-reported smell of smoke from the researcher are a relatively simple and inexpensive methods of assessing SHS exposure^20^. These methods provide an easy tool to provide a snapshot of an environment at a specific point in time. A standardized observational checklist, comprising all the compliance indicators, was adapted from similar studies and was implemented across all venues to improve quality control^20^. The standard indicators of compliance include observed smoking, presence of DSAs, presence of ashtrays and presence of NO SMOKING signs. Additional indicators of compliance such as presence of cigarette butts and the smell of smoke at the venues were also observed in this study. All field workers were trained in entering observation data. Covert data collection was agreed upon based on advice from experts and previous studies on air quality measurement that highlighted the delays and difficulties that an open approach to owners can present^22^. The study protocol was approved by the Ethics Committee of the University of Stirling (Reference number: GUEP494) and KNUST (Reference number: CHRPE/AP/441/18). Data collection was conducted covertly (observation and PM_2.5_ measurements) hence informed consent was not taken, however, researchers carried an official letter during field work describing the study plus evidence of ethical approval and contact details. All the places in which data collection occurred were ‘public places’ and the individuals and the specific locations remain protected by anonymity and confidentiality.

### PM_2.5_ measurements

On entry to each establishment, the researchers purchased a beverage before proceeding to a seat or area as central as possible and away from any doors, windows or obvious potential sources of PM_2.5_ such as open solid-fuel fires or kitchen areas. The researchers aimed to place the monitor on the seat or table level to ensure that sampling was as close as possible to the breathing zone and also tried to ensure that they were not within 1 m of anyone smoking. Air sampling was carried out for a minimum of 30 minutes. This instrument uses a light scattering technique to measure the number of particles in two particle size ranges: more than 0.5 μm and more than 2.5 μm. All data presented in this article relate to particles in the size range between 0.5 μm and 2.5 μm; and were generated as mass concentrations using equations specific to SHS aerosol presented in Semple et al.^23-25^_._ The Dylos devices were switched on to start the logging process at the beginning of each series of visits and were left to measure and log 1-minute particle number concentrations for the duration of the sampling process. SHS assessment was conducted continuously for a period of 30 minutes inside each venue and the device left running between venues to allow PM_2.5_ measurement in outdoor air to provide comparative data. A minimum of 30 minutes of outside air sampling was also undertaken each day in order to provide comparative data on outdoor PM_2.5_ concentrations. Exact entry and exit time for each venue and time spent outside in ambient air were also recorded.

### Data analysis

Study team staff downloaded the air quality data using Dylos Logger software. The Dylos DC1700 measures and records the concentration of particles as described above. Each Dylos device had a specific calibration factor applied from a chamber experiment where measured concentrations of SHS PM_2.5_ were compared with those reported from a calibrated Sidepak AM510 Personal Aerosol Monitor (TSI Inc, MN, USA)^24-26^. Descriptive statistics including the geometric and arithmetic means, standard deviation, minimum, maximum and median were generated for the PM_2.5_ levels across the whole dataset and then subdivided by city, venue type and size of venue using SPSS version 22. Observation data from the standardized checklist were also entered onto an excel sheet, coded and analysed using SPSS version 22. The data were recorded at three time intervals (entry, +15 minutes and +30 minutes) and the mean of the three values was used for the analysis. Descriptive statistics including percentages, proportions, means, standard deviation and medians were generated. The ‘average compliance’ to the smoke-free law was calculated by adding up the values of ‘individual compliance indicators’ and dividing it by the total number of indicators measured.

## RESULTS

### Description of venues

As noted above, a total of 154 venues from three cities were included in the sample. However, two of the venues from Accra and Kumasi had incomplete information, thus 152 venues were included in the final analysis. Out of the 152 venues visited, 62% (n=94) were in Accra, 30% (n=45) in Kumasi and 9% (n=13) in Tamale. About two-thirds (65%, n=94) of the venues were hotels, 15% (n=22) were bars/pubs and 20% (n=29) restaurants. Most of the venues (70%, n=106) were large and permanent structures and could accommodate more than 30 people at a time.

### Compliance with smoke-free laws

The indicators of compliance (presence of DSAs and no-smoking signs, absence of smell of smoke, cigarette butts, ashtrays and any active smoking) were assessed in all 152 venues. NO SMOKING signs were evident in half of the venues (49.5%, n=75) with considerable variations by city: Accra (54.3%, n=51), Kumasi (35.6%, n=16), and Tamale (61.5%, n=8), with DSAs present in less than 10% of the venues (6.6%, n=10) (Table 1). Tobacco smell was recorded in 51 venues (33.6%), and cigarette butts were found on the floor in 19 (12.5%) venues. Only one venue (a hotel in Kumasi) was found to be ‘fully compliant’ with all the indicators of compliance measure in the study (Table 1). More than 90% of the venues visited did not have cigarette or other tobacco products displayed for sale. The total average compliance for all the venues was 63.1% with Accra being the least compliant (59.5%).

**Table 1:** Compliance with specific indicators of smoke-free law in three cities, Ghana

*Indicator*		*Cities*			p*

	*Overall sample (N=152) n (%)*	*Kumasi ^a^ (N=45) n (%)*	*Accra (N=94) n (%)*	*Tamale (N=13) n (%)*	
Presence of no-smoking signage	75 (49.5)	16 (35.6)	51 (54.3)	8 (61.5)	0.007
Presence of DSAs	10 (6.6)	4 (8.9)	5 (5.3)	1 (7.7)	0.509
Absence of smell of smoke	101 (66.4)	39 (86.7)	50 (53.8)	11 (84.6)	0.000
Absence of cigarette butts/ends	133 (87.5)	41 (91.1)	80 (85.1)	12 (92.3)	0.636
Absence of active smoking	125 (82.2)	43 (95.6)	70 (75.3)	12 (92.3)	0.004
Absence of ashtrays	131 (86.2)	40 (88.9)	78 (83.0)	13 (100)	0.567

* p-value based on Fisher’s exact test.

a Only one venue in Kumasi was fully compliant with all the indicators.

Bars and pubs were found to be the least compliant with indicators of smoke-free legislation compared to hotels and restaurants (Table 2).

**Table 2:** Compliance with specific indicators in hotels, bars/pubs and restaurants

*Indicators*	*Type of venue*		

	*Hotels ^a^ (N=101) n (%)*	*Bars/Pubs (N=22) n (%)*	*Restaurants (N=29) n (%)*
Presence of no-smoking signage	55 (54.5)	5 (22.7)	15 (51.7)
Presence of DSAs	4 (4.0)	1 (4.5)	5 (17.2)
Absence of smell of smoke	81 (80.2)	18 (81.8)	13 (44.8)
Absence of cigarette butts/ends	98 (97.0)	8 (36.4)	27 (93.1)
Absence of staff/customer smoking	98 (97.0)	8 (36.4)	19 (65.5)
Absence of ashtrays	94 (93.1)	15 (68.2)	22 (75.9)

a Only one hotel in Kumasi was compliant with all indicators.

### Subjective assessment of SHS

**Figure 1:**
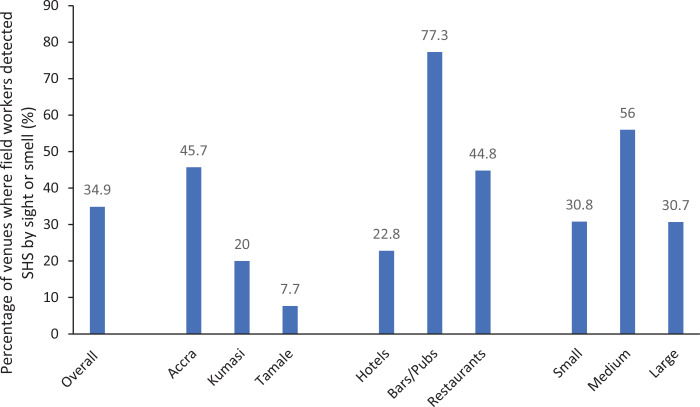
Subjective assessment of SHS by location, venue type and size

The field observers also rated SHS exposure in all the venues as low or zero, medium and high during covert observations and these were converted to binary variables (as present or absent) for analysis. Close to half of the venues in Accra had evidence of SHS exposure, and bars and pubs were more likely to have SHS exposure compared to hotels and restaurants (Figure 1).

### PM_2.5_ measurements

Table 3 shows the PM_2.5_ levels across the different cities, venue type and size. The overall PM_2.5_ concentration (indoors) in all 3 cities was 14.6 μg/m^3^ (median) (Min 5.2, Max 349, IQR 12.9). Overall PM_2.5_ (outdoors) was 12.4 μg/m^3^ (median) (Min 3.8, Max 81.7, IQR 9.4). PM_2.5_ concentrations were higher in Accra compared to Kumasi and Tamale, with bars and pubs having higher indoor PM_2.5_ concentrations than hotels.

**Table 3:** Indoor PM_2.5_ concentrations (μg/m^3^) by city, venue type and size

	*Indoor PM_2.5_*			
	*Median*	*Minimum*	*Maximum*	*IQR*
**City**				
Accra (n=94)	15.8	6.0	349	17.2
Kumasi (n=45)	13.0	5.2	51.3	10.7
Tamale (n=13)	12.5	6.5	23.8	6.5
**Venue type**				
Hotels (n=101)	13.3	5.2	276	9.7
Bars/Pubs (n=22)	21.9	9.0	349	53.4
Restaurants (n=29)	22.0	6.5	335	19.9
Venue size*				
Small	12.6	7.0	66.6	13.1
Medium	22.7	6.1	81.6	31.0
Large	13.9	5.2	349	10.7

* Measurement by how many people can sit in this establishment: 1 – 15 = Small, 16 – 30 = Medium, >30 = Large.

IQR: interquartile range.

Table 4 shows the median and IQR of PM_2.5_ inside, outside and indoor–outdoor grouped by city. The median values in all three cities were below the WHO 24-hour air quality guidance for PM_2.5_ (25 μg/m^3^). It also shows for each city the difference between inside and outside PM_2.5_ concentrations as measured on the day. Positive values indicate that indoor air PM_2.5_ was higher than measured outdoors suggesting the presence of indoor sources of PM_2.5_ emissions.

**Table 4:** PM_2.5_ concentrations (μg/m^3^) measured at indoor and outdoor venues by city

		*Indoor*			*Outdoor*			*Indoor–Outdoor*		

	*Count*	*Median*	*Percentile 25*	*Percentile 75*	*Median*	*Percentile 25*	*Percentile 75*	*Median*	*Percentile 25*	*Percentile 75*
**City**										
Kumasi	45	13.0	8.95	17.6	9.80	8.30	15.89	0.50	-2.80	5.30
Accra	94	15.0	11.7	28.9	14.6	10.5	20.4	2.75	-8.50	11.7
Tamale	13	12.5	7.20	13.7	5.90	5.70	11.7	1.70	1.20	7.70

Table 5 shows results of PM_2.5_ concentrations in locations where smoking was observed (presence of staff/customer smoking, presence of smell of tobacco smoke, cigarette butts, and ashtrays). Venues where smoking was observed had poorer air quality compared to outside, and venues where smoking was not observed had air quality similar to that measured outdoors. Indoor–outdoor concentrations were higher in locations where smoking was observed (6 μg/m^3^) compared to 1 μg/m^3^ where smoking was not observed (p<0.001). In one-quarter of establishments where smoking was observed the indoor PM_2.5_ concentration was at least 25 μg/m^3^ greater than that measured outdoors in that city on the same day.

**Table 5:** PM_2.5_ concentrations (μg/m^3^) in smoking-observed versus smoking-not-observed venues

*Smoking observed*		*Indoor*			*Outdoor*			*Indoor–Outdoor*		

	*Count*	*Median*	*Percentile 25*	*Percentile 75*	*Median*	*Percentile 25*	*Percentile 75*	*Median*	*Percentile 25*	*Percentile 75*
Yes	57	23.80	15.7	61.1	18.2	12.7	30.7	6.00	1.20	25.1
No	95	12.30	9.00	16.0	10.8	8.30	14.0	1.00	-2.80	4.80
*p-value			<0.001			<0.001			<0.001	

* p-value based on multiple linear regression.

## DISCUSSION

The study results demonstrate that close to 60% of the hospitality locations in the three cities were at least partially compliant with the current smoke-free legislation and had no observed smoking during our visit. Findings from other LMICs such as India (where smoking prevalence is much higher) using similar methods for assessing compliance to smoke-free laws recorded higher levels of compliance (>80%) in hospitality locations^27^. This may partly be explained by the development of state- and district-level tobacco control laws alongside strong enforcement of the law in India, which may account for the higher compliance levels. In our study, smoking was observed in about a third of the venues (in areas meant to be smokefree) and less than 1% of the hospitality locations had DSAs and about 50% of the venues had NO SMOKING signage. Interestingly, less than 10% of the venues had tobacco products for sale. Findings from our study clearly indicate that hospitality locations (particularly in Accra) are not fully compliant with current smoke-free legislation several years after the ratification of the FCTC (2004) and passage of the National Tobacco Control Act (2012).

Findings from other countries in Africa such as Kenya with a similar smoke-free policy like Ghana, indicated that smoking occurred in about 85% of hospitality locations^28^. Whereas in Uganda (which has a comprehensive smoke-free law introduced in 2016), observed smoking was present in less than 20% of hospitality locations^29^. The WHO recommends that all countries implement comprehensive smoke-free policies, defined as smoke-free policies with no exemptions for particular venue types or allowances for designated smoking areas as these do not protect against the health harms of secondhand smoke^13^. Reviews in the African region strongly emphasize that the struggle in smoke-free policies in the region are mainly in the areas of implementation and enforcement in addition to other factors such as policy fatigue and limited resources^6,7^. A considerable number of countries in the African region including Ghana have challenges with the enforcement of their smoke-free polices and that the law is continuously breached. Lessons could be learnt from Seychelles, a similar country in Africa, where the compliance to smoke-free laws was impressively high in bars and restaurants after only nine months of the enactment of the smoke-free law^30^. Contributing factors for the situation in Seychelles included a smaller country size (thus requiring fewer resources for implementation), high awareness and knowledge of the smoking ban among hospitality staff, training of hospitality staff on how to enforce the ban, and active enforcement of the ban by venue workers^31^.

The second part of our study objectively assessed SHS exposure by measuring PM_2.5_ concentrations in the hospitality locations within the three cities. Air quality measurement in resource-limited countries in the African Region are rarely carried out and can be expensive and time-consuming^6^. Introduction of low-cost air quality monitors such as the Dylos DC 1700 for measurement of PM_2.5_ has enhanced the quality and quantity of SHS data that are possible to collect and provided evidence needed to strengthen smoke-free protection in low-income settings^21^. In our study, PM_2.5_ measurements in the three cities indicated that venues where smoking was observed had statistically higher PM_2.5_ concentrations compared to those where smoking was not observed. The overall PM_2.5_ concentrations (indoors) in the three cities was 14.6 μg/m^3^ (range: 5.2–348.8) with similar levels in the three cities: Accra (15.5 μg/m^3^), Kumasi (13.0 μg/m^3^), and Tamale (12.5 μg/m^3^). Differences were also observed between the different hospitality venues visited with bars/pubs and restaurants having higher indoor PM_2.5_ than hotels. For this study, we used the WHO recommended 24-h average limit in outdoor air quality of PM_2.5_ of 25 μg/m^3^ as a bench mark^32^. The previous study in Ghana on SHS in 2010 indicated markedly elevated PM_2.5_ (median 553 μg/m^3^; IQR: 259–1038) in smoking venues than in non-smoking venues (median 16.0 μg/m^3^; IQR: 14.0–17.0)^17^. In our study, the median PM_2.5_ measured in smoking venues was higher (23.8 μg/m^3^) compared to nonsmoking venues (12.4 μg/m^3^) (p<0.001). Comparing PM_2.5_ concentrations in hospitality venues in Ghana from 2010 with our results suggests that air quality has markedly improved with PM_2.5_ concentrations having decreased from a median of 553 (pre law) to 14.6 μg/m^3^ in the current study, an almost 97% reduction.

Ghana has made significant progress in terms of improved air quality measurements in hospitality settings. However, public smoke-free law does not fully meet the standards of the WHO FCTC Article 8 (to which Ghana is a Party to); thus, both smokers and non-smokers continue to remain unprotected against SHS in many hospitality locations. There is no risk-free level of SHS and even brief/minimal exposure can cause immediate harm^2,33^. Non-compliance with smoke-free laws among hospitality venues has also been found in other LMICs including those of Africa^21,31^. The results and outcome of this research serve as a basis for discussions on the need to develop specific policies to protect consumers and employees of such premises, and also implement enforcement measures to improve compliance.

### Strengths and limitations

The study’s major strength is the use of a random strategy to sample hospitality venues compared to the previous study in Ghana and several other studies elsewhere that have relied on convenience sampling thus introducing selection bias. Also, the inclusion of a large number of hospitality venues in the three largest cities in Ghana including the use of an objective and subjective assessment of SHS provides a more detailed estimation of SHS exposure in this setting. However, the study has several limitations that need to be noted when considering the study results. First, PM_2.5_ is not specific to SHS and may be generated by other non-smoking sources such as combustion of fuel and traffic pollution, however, our methodology sought to overcome this weakness by measuring outdoor PM_2.5_ to provide comparative data and by presenting the difference between outdoor and indoor concentrations. The results of greater PM_2.5_ concentrations in venues where smoking was observed validate the use of PM_2.5_ as a marker and previous work has also shown high correlation between PM_2.5_ and airborne nicotine in settings where smoking takes place^33^. Other limitations include the study sites limited to the three large urban cities in Ghana and findings may not be representative of all hospitality venues in Ghana. Other weaknesses worth noting are the timing of the data collection that was done from 16:00 to midnight and the months during which the study was conducted (July–September). It may be possible that smoking behavior may differ at different times of the day, the week or month. Lastly, the study is a cross-sectional design hence a causal relationship between smoke-free laws and SHS exposure cannot be implied. However, PM_2.5_ is a well-established marker for SHS and highly correlates with air nicotine.

## CONCLUSIONS

To the best of our knowledge, this is the first study measuring PM_2.5_ concentrations and compliance to the smoke-free law in randomly selected hospitality locations within Ghana’s three largest cities. Smoking was observed in about 37% of the venues and less than one per cent (<1%) of venues were fully compliant with all the measured indicators of compliance. However, there is marked improvement in air quality in these venues compared to earlier studies. Possible reasons for this improvement might be the introduction of the Tobacco Control Act (2012) and the L.I.2247 during this period, which could have led to greater enforcement of smoke-free policies compared to earlier studies and also decreasing smoking prevalence over the years. Fifteen years after the adoption of the WHO FCTC and more than five years after a National Tobacco Control Act, the study identified challenges for complete protection from SHS through legislation. There is still a considerable way to go to increase compliance with the SHS law in Ghana. Efforts are needed to develop an action plan to build on progress towards changing societal norms around smoking in hospitality venues and to ensure greater enforcement of existing smoke-free policy in Ghana.
